# Multi-Omics Approaches to Improve Mitochondrial Disease Diagnosis: Challenges, Advances, and Perspectives

**DOI:** 10.3389/fmolb.2020.590842

**Published:** 2020-11-02

**Authors:** Justine Labory, Morgane Fierville, Samira Ait-El-Mkadem, Sylvie Bannwarth, Véronique Paquis-Flucklinger, Silvia Bottini

**Affiliations:** ^1^Université Côte d’Azur, Center of Modeling, Simulation and Interactions, Nice, France; ^2^Université Côte d’Azur, Inserm U1081, CNRS UMR 7284, Institute for Research on Cancer and Aging, Nice (IRCAN), Centre hospitalier universitaire (CHU) de Nice, Nice, France

**Keywords:** mitochondrial diseases, multi-omics, bioinformatics, diagnosis, personalized medicine

## Abstract

Mitochondrial diseases (MD) are rare disorders caused by deficiency of the mitochondrial respiratory chain, which provides energy in each cell. They are characterized by a high clinical and genetic heterogeneity and in most patients, the responsible gene is unknown. Diagnosis is based on the identification of the causative gene that allows genetic counseling, prenatal diagnosis, understanding of pathological mechanisms, and personalized therapeutic approaches. Despite the emergence of Next Generation Sequencing (NGS), to date, more than one out of two patients has no diagnosis in the absence of identification of the responsible gene. Technologies currently used for detecting causal variants (genetic alterations) is far from complete, leading many variants of unknown significance (VUS) and mainly based on the use of whole exome sequencing thus neglecting the identification of non-coding variants. The complexity of human genome and its regulation at multiple levels has led biologists to develop several assays to interrogate the different aspects of biological processes. While one-dimension single omics investigation offers a peek of this complex system, the combination of different omics data allows the discovery of coherent signatures. The community of computational biologists and bioinformaticians, in order to integrate data from different omics, has developed several approaches and tools. However, it is difficult to understand which suits the best to predict diverse phenotypic outcome. First attempts to use multi-omics approaches showed an improvement of the diagnostic power. However, we are far from a complete understanding of MD and their diagnosis. After reviewing multi-omics algorithms developed in the latest years, we are proposing here a novel data-driven classification and we will discuss how multi-omics will change and improve the diagnosis of MD. Due to the growing use of multi-omics approaches in MD, we foresee that this work will contribute to set up good practices to perform multi-omics data integration to improve the prediction of phenotypic outcomes and the diagnostic power of MD.

## Introduction

Mitochondrial diseases (MD) are rare disorders caused by a deficiency of the mitochondrial respiratory chain, which provides energy to individual cells through oxidative phosphorylation (Munnich and Rustin, 2001). These diseases are extremely heterogeneous, both clinically and genetically, making their diagnosis a real challenge (Gorman et al., 2016). Although mitochondria have their own genome, most proteins involved in their biogenesis are encoded by nuclear genes. Therefore, MD can be caused by pathogenic variants (changes or sequence alterations) affecting either mitochondrial DNA (mtDNA) or nuclear genes (Alston et al., 2017). The advent of high-throughput sequencing (HTS) and its implementation in hospital laboratories has improved the performance of diagnosis which today is based on the analysis of the entire mtDNA and large panels of nuclear genes (Vasli et al., 2012; Plutino et al., 2018). Advances in exome sequencing (WES) and whole genome sequencing (WGS), which is not used routinely, have greatly accelerated the identification of new genes responsible for the disease (Wortmann et al., 2015). However, in one out of two patients, the gene responsible remains unknown.

Genome regulation encompasses all facets of gene expression, from biochemical modifications of DNA to the physical arrangement of chromosomes and the activity of transcription mechanisms. To understand how the different layers of gene regulation act together in pathophysiological contexts, multiple types of data are needed. Lately, several techniques have developed to interrogate this complex process in multiple dimensions (DNA, RNA, proteins, and metabolites), known as “omics.” While these approaches can reveal physiopathological mechanisms in the sample, only the joint use of several omics on the same sample is the key toward the understanding of the associated phenotype. However, there is the need to develop integrative computational approaches to enable the integration of this type of data. The main challenges are to identify models that allow efficient selection of important characteristics and to analyze high-dimensional, scattered and heterogeneous data. To meet these challenges, several algorithms and mathematical structures have been used (Bayesian approaches, matrix factorization methods, multi-step analyses, network-based or machine learning approaches). However, no reference method has been identified yet. Omics data analyzed independently often prove unable to identify genes responsible for MD and explain the complexity of all the molecular phenomena leading to these diseases on their own, thus methods of integrating multiple omics represent a real hope for reducing the diagnostic deadlock for patients with MD.

In this review we will discuss why multi-omics will improve the diagnosis of MD, the few approaches used in the literature on these diseases and their limitations. We will present an up-to-date list of multi-omics algorithms developed in the latest years and we will discuss why these are not employable for MD. A new nomenclature to summarize the different approaches and a data driven interpretation of recent benchmarks will be presented. Finally, we will provide guidelines to develop multi-omics approaches to be used to improve the diagnostic power of mitochondrial diseases.

## Mitochondrial Diseases

Mitochondria are present in all the cells of the body, in variable quantities depending on the energy needs of the organs. MD are due to an energy deficit caused by a dysfunction of the mitochondrial respiratory chain and ATP synthase, which supplies energy to the cells in the form of ATP. MD are a group of rare diseases that are extremely heterogeneous both clinically and genetically. Prevalence is estimated at 1/5,000 births, i.e., about 150 new cases per year in France. MD begin at any age with neonatal forms that are generally more severe than those beginning in adulthood. These diseases therefore affect all organs in isolation or in combination and are generally evolutive. This clinical heterogeneity makes the diagnosis of MD challenging. In addition, many pathological situations and other genetic diseases can lead to a secondary respiratory chain deficiency and there is no single reliable biomarker for MD. The proper functioning of the respiratory chain is dependent on mitochondrial biogenesis and it is estimated that more than 1,500 mitochondrial proteins are involved (MitoCarta2.0). The majority of these proteins are encoded by nuclear DNA (nDNA) but 13 are encoded by mtDNA. Each cell contains 2–10 copies of mitochondrial genome which also codes for 22 transfer RNAs and 2 ribosomal RNAs. As a result, mitochondria are under the control of two genomes, and each clinical presentation results from mutations either in nuclear genes or in mtDNA. In mtDNA encoded disease, a correlation between mutational heteroplasmy level and disease severity is usually observed with a “threshold effect” for disease expression. Heteroplasmy (coexistence of wild-type and mutated mtDNA molecules) is an additional difficulty in the diagnostic process because it requires looking for enzyme deficiency in the affected tissue. This double genetic control also explains why all modes of transmission are observed in these diseases: maternal for mtDNA variants, autosomal dominant, autosomal recessive or X-linked for nuclear genes. *De novo* occurrence is also possible.

The diagnosis of MD is based on the identification of the responsible gene which allows genetic counseling, prenatal diagnosis and sometimes directs treatment choices. Furthermore, it is the first step toward understanding of the disease mechanisms. Today, more than 400 nuclear genes are known to be responsible for MD and the list of candidate genes continues to grow (Craven et al., 2017). Although diagnosis of MD has been completely transformed by the emergence of NGS technologies, to date, more than one out of two patients has no diagnosis in the absence of identification of the responsible gene. Therefore, omics technologies are essential to improve our knowledge of mitochondrial functions (Rahman and Rahman, 2018).

## Omics

### Simple Omics Data Types

The twenty-first century has been marked by the arrival of HTS, such as NGS, which have revolutionized the world of biology. Omics appeared at that time and represent a different strategy, i.e., they study not a single molecule but a set of molecules from the same biological domain, making possible to study biological mechanisms in the globality of living organisms and in the complexity of their interactions.

There are several types of omics (summarized in Table 1). The first to emerge is genomics, which studies the entire genome or the exome. Today, widely used in medical research, it allows the identification of genetic variants that modify the DNA sequence. The study of changes related to the environment or epigenetic factors, is called epigenomics. It improves the functional interpretation of genetic variants found in regions often specific to tissues associated with the disease. Transcriptomics, which is the study of all RNAs products of the genome transcription, allows quantitative and qualitative measurements of transcripts of genes expressed in tissue or cells, identification of new splicing sites and development of knowledge on non-coding RNAs (long RNA, short RNA, circular RNA, etc.). Thanks to the development of mass spectrometry, proteomics (the study of all proteins) and metabolomics (the study of all metabolites: carbohydrates, amino acids, fatty acids, etc.) have been developed, allowing the study of the global interactions of proteins and the quantification of post-translational modifications. The simultaneous quantification of different types of molecules in order to understand the metabolic functions, which in case of deregulation are often involved in diseases, is also possible. Further reviews that explain the different omics technologies in detail are (Hasin et al., 2017; Misra et al., 2019).

**TABLE 1:** Different omics technologies and their characteristics: molecule targeted, omics targeted, sequencing techniques, and their purposes.

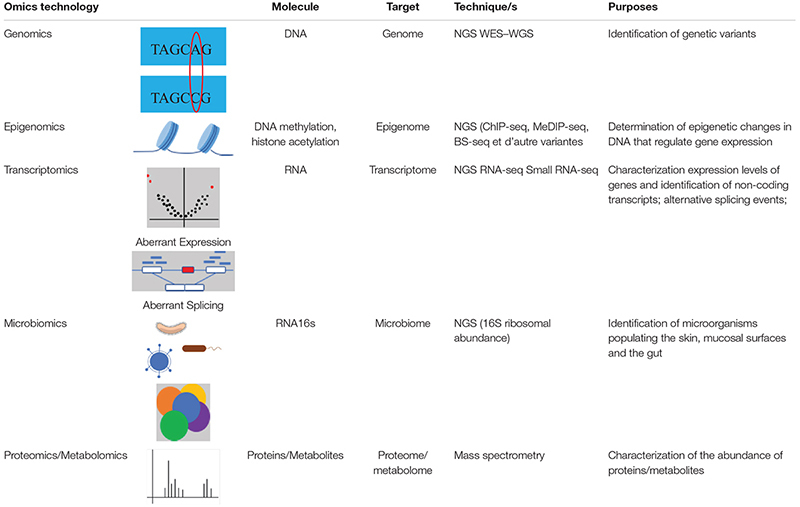

### Simple Omics as a Tool for MD Diagnosis

The search for genes involved in MD is done by NGS and based on the identification of pathogenic nucleotide variants through two techniques: WES (Whole Exome Sequencing) and WGS (Whole Genome Sequencing). WES allows the sequencing of all exons, and exon-intron boundaries that represent 2% of the genome. On average, it allows us to find 20,000 coding variants per individual, of which only 9,000 concern the modification of an amino acid (Stenton et al., 2019). This technique has improved the diagnostic yield of MD estimated at 50% (Stenton et al., 2019). The causes of this low yield are multiple: many VUS, the inability to detect non-coding variants of the genome. In most cases, the potential pathogenicity of VUS cannot be confirmed or invalidated by *in silico* studies. Functional studies in model organisms may be necessary. For an example, the Undiagnosed Diseases Network (UDN),^1^ launched by the NIH, offers platforms such as the Model Organisms Screening Center (MOSC) that uses genetics and biology of *Drosophila melanogaster*, *Caenorhabditis elegans*, and *Danio rerio* to help in the diagnostic of rare diseases. In addition, WGS is used to detect coding and non-coding variants of the genome. However, the number of variants to be interpreted by WGS is very high (about 3 million per individual) and makes difficult its usage in routine diagnosis.

### Toward Multi-Omics Approaches to Improve MD Diagnosis

Omics have thus progressed thanks to technological advances, which have enabled high-powered analysis of biological molecules, with a reduction in cost-effectiveness. Nevertheless, simple omics allow understanding the functioning of biological and pathological processes at a single level, as the different methodologies assess different parts of the complex physiopathology of disease development and progression. However, it is essential to understand the relationships between different molecular entities and their interactions, as well as their role in regulating gene expression (Wani and Raza, 2019).

Despite the wide range of data that can be generated to characterize differences between healthy and diseased cells or tissues, the analysis of a single subset therefore provides an incomplete picture of the underlying biology. More importantly, how do we select the most meaningful types of omics data to be generated, considering the costs and tissue availability?

Whether WES or WGS, they do not allow for the understanding of transcript expression levels or tissue-specific expression that reflects the functionality of a gene and the effect of a variant on it. Thus, the study of the transcriptome by RNA-sequencing (RNA-seq) is a major complement to the WES. Recent studies have shown that the joint use of RNA-seq and WES increases the diagnostic yield of MD by 10% (Kremer et al., 2017). In transcriptomics, three main events are studied and allow to prioritize candidate genes responsible for rare diseases: aberrant transcriptome expression, aberrant splicing and mono-allelic expression (MAE).

**FIGURE 1:**
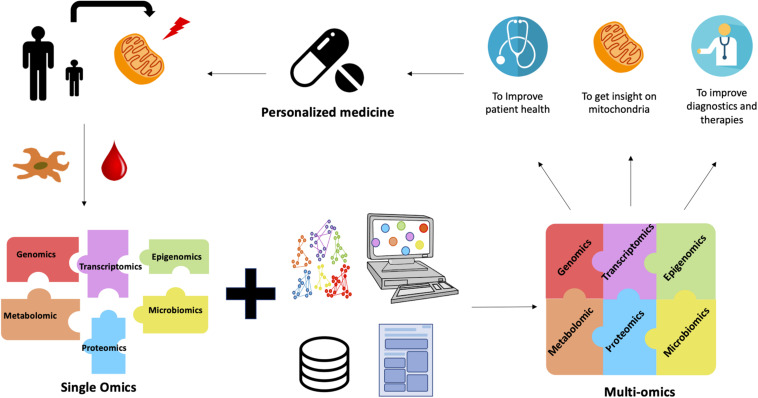
The contribution of multi-omics for MD. Blood or tissue samples (skin or muscle biopsy) are obtained from patients affected by MD. Fibroblasts or myotubes are prepared and cultured from tissue biopsies. Different omics analyses are carried out separately in order to identify the gene(s) responsible for the pathology. However, these analyses are not always conclusive. Despite the validity of the single-omics approach, the complexity of MD limits the success of the diagnosis. This is where multi-omics comes in. Thanks to bioinformatics and the development of algorithms, the integration of data from several different omics analyses is possible and leads to better results. Multi-omics combined with databases and literature provide with a better understanding of mitochondrial diseases. Their diagnosis is therefore improved and a personalized treatment can be proposed for each patient.

Another omics tool that can be used to determine the impact of these variants is proteomics. This omics technique provides a functional validation of variants and completes the outliers detected in transcriptomics. A significantly reduced protein level in a sample compared to other samples or controls is a strong evidence of the presence of a variant that is responsible for this decrease (Stenton et al., 2019). This work has paved the way for multi-omics approaches in the study of MD (Figure 1). Instead of using the results of the omics analyses separately, better results are obtained if the results of several different omics analyses are cross-referenced. The use of existing databases, bioinformatics and literature, in addition to multi-omics improves the understanding of mitochondrial diseases in order to improve the health of patients through personalized treatment.

### Mitochondrial Databases

Since their first employ, omics techniques have generated a significant amount of complex and voluminous data. These data are available on online databases. There are many mitochondrion-specific ones such as MitoCarta (Calvo et al., 2016), MITOMAP (Kogelnik et al., 1996), MitoMiner (Smith and Robinson, 2018), or HmtDB (Clima et al., 2017) that contain data on the mitochondrial genome and its variants. An exhaustive list of these databases is provided in Table 2.

**TABLE 2:** Non-exhaustive list of available mitochondrial databases.

Database	Content	Web site	Last update
**Dedicated mtDNA databases**			
MITOMAP	A compendium of polymorphisms and mutations in human mitochondrial DNA	https://www.mitomap.org	2020
MSeqDR	List of mitochondrial disease with associated symptoms, genes and variants	https://mseqdr.org	2020
HmtVar	Variability and pathogenicity information about mtDNA variants	https://www.hmtvar.uniba.it	2019
HmtDB	Human mitochondrial genome sequences annotated with population and variability data	https://www.hmtdb.uniba.it	2019
EMPOP	Collection of mtDNA haplotypes from various world populations	https://empop.online/	2019
MitoMiner	Mitochondrial localization evidence and phenotype data for mammals, zebrafish and yeasts	http://mitominer.mrc-mbu.cam.ac.uk	2018
MitoBreak	Curated datasets of mtDNA rearrangements.	http://mitobreak.portugene.com/	2017
MitoProteome	Object-relational mitochondrial gene/protein sequence database and annotation system	http://www.mitoproteome.org/	2016
Human MitoCarta2.0	Inventory of 1,158 human and mouse genes encoding proteins with strong support of mitochondrial localization	http://www.broadinstitute.org/pubs/MitoCarta	2015
mtDB	Clinical features of mitochondrial disease	http://mitodb.com/	2015
MitoP2	Human, Mouse and Yeast proteins with mitochondrial localization	https://omictools.com/mitop2-tool	2009
Human Mitochondrial Protein Database	Comprehensive data on mitochondrial and human nuclear encoded proteins involved in mitochondrial biogenesis and function	https://bioinfo.nist.gov/	2007
mtSNP	Mitochondrial SNPs associated with different conditions (age, Alzheimer, Parkinson, obesity)	http://mtsnp.tmig.or.jp	2006
**Databases including mitochondrial data**			
ClinVar	Links between variations and human phenotypes	https://www.ncbi.nlm.nih.gov/clinvar/	2020
OMIM	Online catalog of human genes and genetic disorders	https://omim.org/	2020
ClinVar Miner	Interpretation data for ClinVar variants	https://clinvarminer.genetics.utah.edu/	2020
Human Gene Mutation Database (HGMD)	Collection of germline mutations in nuclear genes associated with human hereditary diseases	http://www.hgmd.cf.ac.uk/	2017

*Table listing the databases containing information on mitochondria and mitochondrial diseases, links to web site databases and the year of their last update.*

However, the volume of data creates considerable challenges to enable meaningful conclusions to be drawn. To date, MITOMAP contains 14,431 mtDNA variants, MitoMiner and MitoCarta contain approximately 1,157 human and mouse genes encoding mitochondrial proteins. There are also databases associating clinical data or pathologies with genetic variants such as OMIM (Amberger et al., 2015), ClinVar, ClinVar (Landrum et al., 2013), Miner (Henrie et al., 2018), or HGMD (Stenson et al., 2017; Bris et al., 2018).

### Other Public Omics Databases

Multi-omics data broadly cover the domains of “-omes” and can provide useful biological information at several levels and thus help to understand the mechanisms of pathologies, contribute to diagnosis, prognosis and potential therapeutic interventions (Urbanski et al., 2019). There are many examples of massive data production in specific applications for human diseases. Most of these projects are publicly funded and are collected in open access online databases (listed in Table 3). However, much of this data is either not used or not fully analyzed, creating a great disparity between the generation and use of data. Moreover, this huge amount of data does not translate into knowledge and it is not currently applied in clinical practice.

**TABLE 3:** Non-exhaustive list of the main public omics databases (listed alphabetically).

Databases	Types of data	Number of sites	Number of samples	Links
ArrayExpress	Healthy + Diseases	5 types of molecules	27,462	https://www.ebi.ac.uk/arrayexpress/
CCLE	Cell line cancers	39 tissues	1,457	https://portals.broadinstitute.org/ccle
ColPortal	Healthy + Diseases	48	253	https://colportal.imib.es/colportal/index.jsf
CPTAC	Cancers	10 tissues	772	https://proteomics.cancer.gov/programs/cptac
dbGAP	Healthy + Diseases	1,513 studies	2,935,530	https://www.ncbi.nlm.nih.gov/gap/
ENCODE	Healthy + Diseases	94	7,536	https://www.encodeproject.org
GDC	Cancers	67 tissues	84,031	https://portal.gdc.cancer.gov
GEO	Healthy + Diseases	55,176 entries	1,957,921	https://www.ncbi.nlm.nih.gov/geo/browse/
gnomAD	Healthy + Diseases	9 populations	71,702	https://gnomad.broadinstitute.org
GTEx	Healthy	54 tissues	17,382	https://www.gtexportal.org/home/
HMDB	Healthy + Diseases	114,184 metabolites	25,000	https://hmdb.ca
ICGC	Cancers	22 tissues	24,289	https://icgc.org
METABRIC	Breast cancers	1 tissues	2,509	https://www.cbioportal.org/study/summary?id=brca_metabric
MGnify	Healthy + Diseases	20	127,417	https://www.ebi.ac.uk/metagenomics/
Omics discovery index	Healthy + Cancers	30 tissues	92,846	https://www.omicsdi.org
PCAWG	Cancers of ICGC	20 tissues	2,793	https://dcc.icgc.org/pcawg
PDB	Healthy + Diseases	5 types of polymers entities	47,552	https://www.rcsb.org
Roadmap epigenomics	Healthy + Diseases	310	127	https://egg2.wustl.edu/roadmap/web_portal/index.html
TARGET	Pediatric cancers	16 tissues	6,197	https://ocg.cancer.gov/programs/target
TCGA	Cancers	30 tissues	11,315	https://www.cancer.gov/about-nci/organization/ccg/research/structural-genomics/tcga
1000 genomes	Healthy Diseases	26 populations	2,504	https://www.internationalgenome.org/home

*For each database, we report the type of data (healthy or Diseases), the number of site (tissues, populations, experiments) available, the number of human samples available, and the links to web databases. Numbers are only given for organism Homo Sapiens. CCLE, Cancer Cell Line Encyclopedia; CPTAC, Clinical Proteomic Tumor Analysis Consortium; dbGAP, database of Genotypes and Phenotypes; ENCODE, Encyclopedia of DNA Elements; GDC, Genomic Data Commons; GEO, Gene Expression Omnibus; gnomAD, genome aggregation database; GTEx, Genotype-Tissue Expression; HMDB, The Human Metabolome Database; ICGC, International Cancer Genome Consortium; METABRIC, Molecular Taxonomy of Breast Cancer International Consortium; PCAWG, Pan Cancer Analysis of Whole Genomes; PDB, Protein Data Bank; SRA, Sequence Read Archive; TARGET, Therapeutically Applicable Research To Generate Effective Treatments; TCGA, The Cancer Genome Atla.*

Finding an appropriate method for data integration and interpretation is often complicated because the data are heterogeneous, large and composed of several variables. Although there are several methods for multi-omics analysis, choosing the most appropriate method for each dataset is quite difficult. To meet these needs, the development of a new classification of multi-omics analytical methods is fundamental.

## The ERA of Multi-Omics

### New Classification of Multi-Omics Integration Methods

Multi-omics methods are emerging as valuable tools for understanding the functioning of the mitochondria. Data integration is defined as a process by which data from different sources are combined statistically to make large-scale conclusions about a disease and to obtain a comprehensive view of biological processes. Omics integrative approaches increase the reliability of a biological discovery if it can be validated by concordant omics signatures (genomics, transcriptomics, and proteomics) (Maldonado et al., 2019).

The classification of methods for multi-omics integration is currently quite complex because each article proposes its own classification, making the choice of their use complicated. Subramanian et al. (2020) classify the different methods into six categories, but also into three different case studies to answer biological questions. Huang et al. (2017) detail integration methods by examining unsupervised, supervised, and semi-supervised algorithms. Rappoport and Shamir (2018) classified the methods into three categories: early, intermediate and late integration.

**FIGURE 2:**
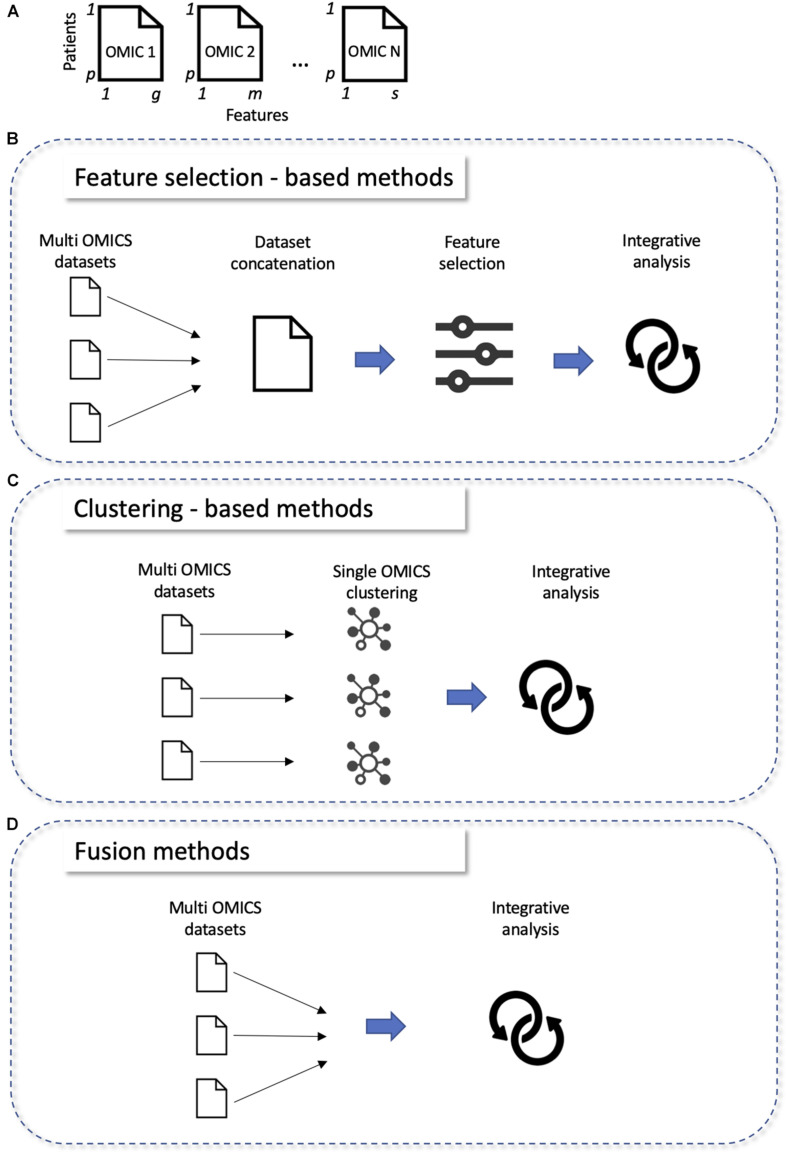
Classifications of multi-omics integration methods. **(A)** Structure of omics data before integration: each omics (OMIC N) correspond to a matrix with p lines (patients) and g, m, or s columns (features). **(B–D)** New classification of omics integration tools: “Feature selection,” “Clustering,” “Fusion methods,” **(B)** single omics are integrated together, then a step of features selection is employed; **(C)** clustering is done at single omics level, then clusters are integrated together; **(D)** single omics are directly integrated together.

All these classifications are mainly based on the type of algorithm, making difficult to choose which method to use depending on the characteristics of the dataset. For this purpose, we propose a new classification with three categories based on the way the methods analyze the data: “feature selection,” “clustering,” and “fusion” (Figure 2, a list of methods belonging to each classification is reported in Table 4).

**TABLE 4:** Non-exhaustive list of the main integration tools and methods grouped by the new classification and sorted by category, then by year and by decreasing number of citations.

Category	Methods	References	Quotes	Supervised/Unsupervised	Techniques	Types of OMICS	Advantages/Disadvantages
Feature selection	MOFA	Argelaguet et al., 2018	97 (2018) 4 (2017)	Unsupervised	Bayesian	All types	Works only for linear relationships
Feature selection	mixOmics	Rohart et al., 2017	401	Supervised and unsupervised	Multivariate/Matrix factorization	All types	High-performance, but noise-sensitive classification
Feature selection	rMKL-LPP	Speicher and Pfeifer, 2015	70	Unsupervised	Multiple kernel	All types	Different choices of reduction methods, more flexibility and comparable results
Feature selection	Joint Bayes Factors	Ray et al., 2014	50	Unsupervised	Matrix factorization/Bayesian	All types	Student’s test, assumes a close relationship between the different levels of data, linear relationship between latent space and observation space
Feature selection	JIVE	Lock et al., 2013	197	Unsupervised	Matrix factorization	All types	Processes only Gaussian distribute data. Sensitive to noise and outliers
Feature selection	CONEXIC	Akavia et al., 2010	235	Unsupervised	Network-based/Bayesian	Transcriptomics/Genomics	Score-guided search to identify a combination of genes Information on the role of associated factors and genes
Feature selection	IntergrOmics	Lê Cao et al., 2009	217	Unsupervised	Regression-based	All types	Exploration by canonical correlation analysis (maximizes correlation) and by least squares (maximizes covariance)
Feature selection	MFA	de Tayrac et al., 2009	51	Unsupervised	Multivariate/Matrix factorization	All types	Better performance on simulated data (=2 types of omics) Little affected by noise Gives a balanced representation of individual and common structures
Feature selection	jActive Modules	Ideker et al., 2002	418	Supervised	Network-based	All types	External contribution of significance measurements on genes Subjected to the gene interaction network High performance for finding the hidden directory on the control channels
Clustering	iOmicsPASS	Koh et al., 2019	5	Supervised	Network-based	All types	Predictive feature across molecular interactions Very limited search space Good prediction error rate Very suitable for small sample sizes
Clustering	SNF	Wang et al., 2014	622	Unsupervised	Network-based	All types	A deeper and more global vision Noises of weak similarities are eliminated, and strong similarities are added. Flexible, few constraints on the input files
Clustering	iClusterPlus	Mo et al., 2013	209	Unsupervised	Matrix factorization/Bayesian	All types	Different modeling assumptions (logistic, linear, logit, fish.) No non-negative constraints, but need to preselect features Takes into account categorical + continuous variables (absent in iCluster) Difficult statistical inference, complexity of the calculation and very time consuming
Clustering	BCC	Lock and Dunson, 2013	138	Unsupervised	Bayesian	All types	Assumes that the data is represented normally
Clustering	MDI	Kirk et al., 2012	114	Unsupervised	Bayesian	All types	Flexible, can group to a single dimension in multiple data
Clustering	PARADIGM	Vaske et al., 2010	402	Unsupervised	Network-based/Bayesian	All types	External classification (NCI, PID) Does not take into account interactions (independently measured pathways)
Fusion method	iGC	Lai et al., 2017	8	Supervised	Student test	Transcriptomics/Genomics	Fast, easy to use
Fusion method	MethylMix	Gevaert, 2015	55	Unsupervised	Multi-staged	Transcriptomics/Genomics	Fast, easy to use Large cohort size
Fusion method	MCIA	Meng et al., 2014	135	Unsupervised	Matrix factorization	All types	Same set of samples, but not necessarily the same molecular features.
Fusion method	NMF	Zhang et al., 2012	159	Unsupervised	Matrix factorization	All types	Slow, large memory space. Input matrix values only positive, and normalization step
Fusion method	PSDF	Yuan et al., 2011	55	Unsupervised	Bayesian	Transcriptomics/Genomics	Binary state in function if the elements match or not (only keeps the matches) Noise reduction selection and 2 types of data entry, reducing the flexibility of cross-platform analysis
Fusion method	PMA (mCCA, sCCA, ssCCA…)	Witten and Tibshirani, 2009	213	Unsupervised	Canonical correlation analysis	All types	Several versions of CCA (canonical correlation analysis) Powerful
Fusion method	SDP/SVM	Lanckriet et al., 2004	240	Supervised	Multiple kernel	All types	Based on the similarity, quite effective Prototype does not include programming software

*We report their reference article, the number of citations in the last 5 years, the principal method/algorithm and whether they are supervised or not, the types of OMICS on which they can be applied, as well as their advantages and/or disadvantages.*

In the next paragraphs, the main multi-omics integration techniques existing in the literature will be presented according to our new classification with examples of the most commonly used algorithms.

#### Feature Selection Methods

Feature selection methods take the results of simple omics data, concatenate them, and perform variable selection using different techniques (Figure 2B). The most cited methods are mixOmics (Rohart et al., 2017), JIVE (Lock et al., 2013), CONEXIC (Akavia et al., 2010), jActive Modules (Ideker et al., 2002), and IntergrOmics (Lê Cao et al., 2009). For example, MixOmics (Rohart et al., 2017) allows the integration of multi-omics datasets using different methods such as PCA and partial least squares regression. MixOmics can address both disease subtype and biomarker prediction. It is very often used in the context of cancers. Another example, multi-omics factor analysis (MOFA) (Argelaguet et al., 2018) is an unsupervised method for finding the main sources of variation in multi-omics data sets. MOFA allows a variety of downstream analyses, including sample subgroup identification, data imputation and outlier detection. It was applied to a cohort of patients with chronic lymphocytic leukemia (Subramanian et al., 2020). It identified the main factors of variability between patients, which improved the interpretation of data and facilitated the definition of predictive models of clinical outcomes.

#### Clustering Methods

Clustering methods use clustering techniques to identify clusters on single omics data (Figure 2C). In classification problems, data can first be transformed through similarity or covariance before joining them. This category preserves the specific properties of the data types and allows the addition of external classification (biological, metabolic pathways) to improve the performances. Methods such as Similarity network fusion (SNF) (Wang et al., 2014), iClusterPlus (Mo et al., 2013), or Pathway Recognition Algorithm using Data Integration on Genomic Models (PARADIGM) (Vaske et al., 2010) are widely used and are applicable to all types of omics. SNF method (Wang et al., 2014) is a network approach to integrate multi-omics data using a network fusion method. The advantage of this method is that weak connections disappear with iterations, while strong connections are reproduced until convergence. It was tested on data from patients with different glioblastoma subtypes. PARADIGM is another example and allows the activities of patient-specific biological pathways to be inferred from multi-omics data (Vaske et al., 2010).

#### Fusion Methods

Fusion methods directly take all available single omics data and merge them (Figure 2D). This preserves the properties of each specific data type, as we are able to analyze each type individually, however their mutual relationship are not known, which can lower down the performances of the final model. This is why the methods in this category in general can only be applied to two types of omics (transcriptomics and genomics) and are less cited than methods in the other two categories. Methods such as non-negative matrix factorization (NMF) (Zhang et al., 2012), Penalized Multivariate Analysis (PMA) (Witten and Tibshirani, 2009), Semidefinite Programming/Support Vector Machine (SDP/SVM) (Lanckriet et al., 2004) appear to be equally effective. Multiple co-inertia analysis (MCIA) (Meng et al., 2014) is another example of fusion method that can be used for determining co-relationships between datasets (such as gene expression, microRNA expression, protein expression).

### Comparison Between Integration Methods

How can the performances of these methods be evaluated? To answer this question, the researchers conducted studies on several data sets to show the performance and limitations of the different methods. Here we will discuss the main findings of these valuable benchmarks.

The paper by Huang et al. (2017) demonstrated that the SNF, cluster method, achieved the highest performances in the majority of the tests (9/22) and proved to be the most robust especially when the complexity of the data increase. Their analysis also showed that the integration of more and more omics data allows a better classification of samples and increases the precision. However, this process can add noise and decrease the signal strength of the omics data, which negatively influences the results.

Tini et al. (2017) compare five methods belonging to the three categories (feature selection: JIVE and Multiple factor analysis (MFA); clustering: SNF; fusion: mCCA). According to the authors, none of the methods is the most efficient one and they all need to be improved, for example by adding information on the relationships between different data in omics, which could reduce false positives while improving the relevance of true molecular interactions (Tini et al., 2017).

Rappoport et al. demonstrated that in most of the cases the rMKL-LPP (Rappoport and Shamir, 2018), feature selection method, achieved the best results in terms of clinical enrichment, and outperformed all but the mCCA (feature selection) and MultiNMF (fusion) methods in terms of survival. Although the high performances of mCCA and MultiNMF are remarkable (Speicher and Pfeifer, 2015), they should not always be preferred because of multiple factors such as: complexity of multi-omics data, noise due to sequencing technique, medical issues, etc.

Overall, in case of complex biological data (several subtypes, several omics, low signal), it is recommended to choose a method from the feature selection category that performs a feature selection step to attenuate the noise.

### Multi-Omics Approaches for Mitochondrial Diseases

To date, algorithms for multi-omics data integration, have very little application to MD because they are developed to be used on a large number of patients and which are not applicable on rare disorders as MD.

The limited number of patients is not the only difficulty in applying existing algorithms for multi-omics integration to MD. MD are rare and heterogeneous and the causative variant(s) are usually unique or “private” for each patient (or family). They require a methodology that identifies unique signatures making difficult to apply most of multi-omics methods available because they are more suitable to identify common signatures.

The specificity of MD has led scientists to reinvent new approaches to integrate multi-omics data from MD patients to improve their diagnosis. In this paragraph, the first studies that have demonstrated the feasibility and usefulness of multi-omics approaches for MD will be described.

Kremer et al. decided to integrate WES data with RNA-seq data to identify variants responsible for MD for a cohort of 48 patients (Kremer et al., 2017). They have developed a pipeline to detect three main causes responsible for variants: aberrant transcript expression, aberrant splicing and MAE. Thanks to their bioinformatics approach created *ad hoc*, they found one aberrantly expressed gene, five aberrant splicing events and six mono-allelically expressed variants. This approach resulted in the diagnosis of 5 patients from the 48-patient cohort with undiagnosed MD and the identification of a candidate gene for 36 other patients.

To improve the results and design a tool that can be used on other datasets, most of the same authors as this pioneer work, developed the OUTRIDER (OUTlier in RNA-seq fInDER) method that identifies “outlier” genes that are aberrantly expressed in the entire cohort (Brechtmann et al., 2018). OUTRIDER is based on the use of auto-encoders, a deep learning approach. However, OUTRIDER does not yet allow the integration of multi-omics data but only RNA-seq data from different platforms (e.g., patients vs. Genotype Tissue Expression database). Further development of this promising method would allow the integration of multi-omics data for MD in the future.

Stenton et al. (2019) also used an *ad hoc* bioinformatics approach. They analyzed the inconclusive cases in the WES samples by running transcriptomics and proteomics analyses in parallel. They tracked the impact of a variant on the abundance of transcripts and their sequences during translation and vice versa by tracing the aberrant expression and splicing back to the responsible protein.

Gonorazky et al. (2019) developed a pipeline based on transcriptome analysis. They extracted total RNA from muscle and skin biopsy samples. They focused on a panel of 132 genes known to be involved in neuromuscular diseases. The rest of the pipeline is very similar to the one put in place by Kremer et al. for RNA-seq data. The main difference is that Gonorazky et al. identify variants on transcriptome data. Using this strategy, they solved 36% of the inconclusive WES and/or gene panel cases.

These first attempts at multi-omics data integration, although promising, need to be improved. In particular, there is the need to implement algorithms that are reproducible and widely employable beyond *ad hoc* approaches in order to standardize the analysis of multi-omics data for MD. For example, the WeiGhted Correlation Network Analysis (WGCNA) (Langfelder and Horvath, 2008) method that integrates genomic, transcriptomic and recently metabolomic data could be applied to MD to analyze multi-omic data, with no constraints on the number of patients needed. This tool allows to explore metabolic pathways in order to identify which pathway is deregulated and therefore which genes are involved.

Bioinformatics tools particularly useful in MD include SAVNet (Splicing-Associated Variant detection by NETwork modeling) (Yamada et al., 2019). It allows the variants obtained to be cross-referenced with aberrant splicing sites to determine whether certain variants are responsible for these events that subsequently cause changes in gene expression and protein abundance. It is therefore conceivable to integrate this tool into the pipeline set up by Kremer et al. to integrate WES and RNA-seq data.

## Methodological Challenges in the Fields of Multi-Omics Integration

Although many data are public, they cannot be integrated simply or directly into a mathematical framework or statistical model. The integration of these data to obtain a global understanding of biological processes and diseases presents particular challenges: the underlying heterogeneity of the omics data, the large size of the data leading to intensive analysis of the calculations, and the lack of studies to prioritize the various tools (Subramanian et al., 2020). One of the main limitations of integrative approaches is related to dimensionality, because even though several layers allow a more complete understanding of the biological system, the dimension of the problem increases (Bersanelli et al., 2016). Clinical information is a dimension that could also enhance the interpretation of multi-omics data.

In addition to these challenges, one of the major obstacles is the non-standardization of data formats in different technologies. Most multi-omics integrative analysis tools require the data to be in specific formats (Figure 2A), so individual omics data must be pre-processed. The pre-processing stage includes data filtering, systematic standardization, batch effect removal and quality control. It becomes imperative to use these pre-processing steps carefully as they have a considerable influence on the integrative analysis. But these data are difficult to transform into machine-readable format, often because of the lack of uniform data representations, the absence of standard nomenclature for designating biological entities (genes, proteins, …), incorrect data annotation and ambiguous vocabulary (Subramanian et al., 2020).

The availability of methods that are not specific to a type of omics will allow the extension of integration applications to approaches that are still little addressed by specific methods (proteomics, metabolomics) (Bersanelli et al., 2016). A crucial factor for ergonomics and dissemination of methods is to have well-documented and easy-to-use software. However, there are still cases where software is not provided.

Finally, the main key to any integrative analysis is the right choice of method to answer the question of biological or medical interest. There are many studies that provide a comparative analysis of integration tools (as discussed in the paragraph “Comparisons between integration methods”), but they are not comprehensive enough in terms of the choice of tools and the biological context. Further studies of this type are needed to guide the community in gaining a better understanding of the wide range of tools.

## Beyond MD: The Integration of Omics in Personalized Medicine

A new era of personalized medicine has arrived, offering a project of individualized care with treatment and medical management targeted and adapted to each patient. The continuous improvement of broadband technologies facilitates this process by transmitting detailed information about the human body (Chen and Snyder, 2012). The integration of omics allows the pathophysiological status of the patient to be reflected at the time of sample collection, thus providing a better understanding of the biology of pathology and drug response (Rotroff and Motsinger-Reif, 2016).

The personalized approach in omics catalyzes precision medicine on two levels. For diseases and biological processes whose mechanisms are still unclear, it will facilitate research that would greatly advance our understanding; and when mechanisms are clarified, individualized care can be provided through health surveillance, preventive medicine and personalized treatment. This approach also facilitates the development of other important health-related fields, such as nutritional systems biology, which studies personalized diet and its relationship to health from a systems perspective (Chen and Snyder, 2012).

With the rapidly declining costs of omics technologies, we expect an increasing number of applications in the development of personalized medicine and in many aspects of health care. This will considerably improve the price charged to patients and reduce the cost of care for the general public. Scientists, governments, pharmaceutical companies and patients should work closely together to ensure the success of this transformation. As part of health surveillance, the iPOP (integrative personal omics profiles) tool (Chen and Snyder, 2012) is used to track individual genomics, transcriptomics, proteomics, metabolomics and autoantibody profiles. This technology is successfully used to identify the health and disease states of a single individual, which shows the real interest in personalized medicine. These approaches are currently underdeveloped but offer great hope for the management and prevention of complex diseases.

## Discussion and Conclusion: Open Challenges and Future Directions

Progress in the NGS has reduced the number of patients in diagnostic impasse, but it is still not enough. Multi-omics approaches are very promising for improving diagnostic performances, but several problems remain to be solved. They are generally developed for cancer research where large numbers of samples are available, which is not the case for MD. Therefore, there is a need to develop multi-omics approaches applicable to small cohorts. Moreover, in oncology, these methods look for common signatures, whereas for MD, there are mainly “private” signatures for one patient or one family, i.e., an altered gene for a patient. However, it could be possible to identify common signatures for small groups of patient, e.g., deficiency of a specific complex of the respiratory chain.

Another challenge is to develop databases specific to MD. For rare diseases, very few patients are affected, so few data are available and especially very few patients share the same pathogenic variant. Data from a single hospital are not sufficient and the establishment of interoperable national and European clinical-biological databases would allow us to expand the available cohorts and accelerate the knowledge of these diseases. Several initiatives have been set up, including the RD-Connect project. Funded by the European Union since 2012, RD-Connect is developing data sharing mechanisms and tools for omics and bioinformatics analysis that are incorporated into an integrated platform linking patient registries, biobanks and clinical bioinformatics data into a central resource for rare disease research (Johnston et al., 2014).

In the future, international and interdisciplinary collaborations are essential to develop more effective tools and share data to fight the diagnostic impasse and improve patient management.

This review has therefore enabled us to develop a new classification by summarizing the main methods of multi-omics integrations, which will benefit the entire scientific community by simplifying their choice of a method adapted to each type of data set.

In conclusion, multi-omics is nowadays evolving in bioinformatics and will soon go beyond the use of single omics in biological and medical research to obtain a better understanding of human diseases, to develop approaches for predicting outcomes, biomarker discoveries and molecular signatures (Figure 1).

Nevertheless, in the future several points remain to be developed. First, more comparative analyses will be needed to assess the performance of tools in contexts other than cancer, as this will allow the selection of the right tool based on the dataset, even if one tool may not always be preferred. Reference data sets should also be developed using simulation tools, which will allow for more accurate estimation of false positives and false negatives. Two other points need to be developed. On one hand, the development of tools that can be used on small cohorts and capable of managing more variables than patients. On the other hand, the development of databases for healthy individuals that will be used as a control to calibrate the tools.

Collaboration between scientists from different fields is also essential for the integration of multiple layers of information. This superimposition of information is very useful for elucidating how pathological processes occur, as well as for the development of new therapeutic interventions.

## Author Contributions

JL and MF performed bibliographic research, wrote the manuscript, and prepared figures and tables. SiB and VP-F conceived the review and wrote the manuscript. SA-E-M and SyB contributed to the manuscript and critical interpretation. All the authors read and approved the final version of the manuscript.

## Conflict of Interest

The authors declare that the research was conducted in the absence of any commercial or financial relationships that could be construed as a potential conflict of interest.

## Footnotes

1 https://undiagnosed.hms.harvard.edu/
